# Polyphenols in Almond Skins after Blanching Modulate Plasma Biomarkers of Oxidative Stress in Healthy Humans

**DOI:** 10.3390/antiox8040095

**Published:** 2019-04-10

**Authors:** C.-Y. Oliver Chen, Paul E. Milbury, Jeffrey B. Blumberg

**Affiliations:** 1 Antioxidants Research Laboratory, Jean Mayer U.S. Department of Agriculture Human Nutrition Research Center on Aging at Tufts University, Boston, MA 02111, USA; oliver.chen@tufts.edu; 2 Friedman School of Nutrition Science and Policy, Tufts University, Boston, MA 02111, USA; tollers@verizon.net

**Keywords:** almond skins, bioavailability, waste by-products, flavonoids, oxidative stress, human

## Abstract

Almond skins are a waste byproduct of blanched almond production. Polyphenols extracted from almond skins possess antioxidant activities in vitro and in vivo. Thus, we examined the pharmacokinetic profile of almond skin polyphenols (ASP) and their effect on measures of oxidative stress. In a randomized crossover trial, seven adults consumed two acute ASP doses (225 mg (low, L) or 450 mg (high, H) total phenols) in skim milk or milk alone. Plasma flavonoids, glutathione peroxidase (GPx), glutathione (GSH), oxidized GSH (GSSG), and resistance of low- density lipoprotein (LDL) to oxidation were measured over 10 h. The H dose increased catechin and naringenin in plasma, with maximum concentrations of 44.3 and 19.3 ng/mL, respectively. The GSH/GSSG ratio at 3 h after the H doses was 212% of the baseline value, as compared to 82% after milk (*p* = 0.003). Both ASP doses upregulated GPx activity by 26–35% from the baseline at 15, 30, 45, and 120 min after consumption. The in vitro addition of α-tocopherol extended the lag time of LDL oxidation at 3 h after L and H consumption by 144.7% and 165.2% of that at 0 h compared to no change after milk (*p* ≤ 0.05). In conclusion, ASP are bioavailable and modulate GSH status, GPx activity, and the resistance of LDL to oxidation.

## 1. Introduction

Nuts, including almonds, are a nutrient-dense food containing minerals, vitamins, unsaturated fats, protein, and fiber [1]. An association between nut consumption and a reduced risk of cardiovascular disease (CVD), certain types of cancer, type 2 diabetes mellitus, and all-cause mortality has been reported in the ever-growing literature [2,3,4,5]. Due in part to the recognition of these health benefits, nut consumption has been gradually increasing around the globe since 2006 [6]. Among tree nuts, almonds are the most consumed. Commercially, almonds can be found in the shell, shelled, and peeled, with peeled almonds (devoid of the seed coat or skin) being commonly used as a raw material for confectionary and bakery applications. In the industrial setting, peeled almonds are produced via hot water blanching, a process which generates blanched water and almond skins, byproducts that are sometimes characterized as environmental pollutants. Almond skins are generally utilized in livestock feed and composting [7,8]. To promote sustainable agricultural and food systems, almond skins, which are rich in fiber and phenols, have the potential for valorization in functional foods, nutraceuticals, and/or food additives [9,10,11,12].

Almond skins have been recognized as a potential functional ingredient in foods due to their antioxidant polyphenols and prebiotic fiber [13,14,15]. Mandalari et al. [14] reported that almond skins have a favorable probiotic index (a comparative relationship between the growth of beneficial bacteria and less desirable ones), comparable to that of fructo-oligosaccharides. The profile of polyphenolics in almond skins includes flavonoids, phenolic acids, and proanthocyanins [16,17]. As ubiquitous secondary metabolites, polyphenols are found in relatively high concentrations in the seed coats or skins as phytoalexins where they act to protect nutrients in the seed kernel from oxidation and microbial action until germination [18]. Meta-analytic studies are largely consistent in showing that foods rich in flavonoids ameliorate CVD risk factors and reduce all-cause mortality outcomes [19,20,21]. The health benefits of flavonoids have been ascribed to their multifaceted bioactions, such as antioxidation, anti-inflammation, vasodilation, anti-hypertension, platelet function, insulin sensitization, and cholesterol reduction [18,22]. In addition, polyphenols present in almond skins have been reported to display antimicrobial effects against a range of food-borne pathogens, such as *Listeria monocytogenes*, *Staphylococcus aureus*, and *Salmonella enterica* [23].

We have previously reported that almond skin polyphenols (ASP) are bioavailable and protect low-density lipoprotein (LDL) against oxidation in hamsters [24]. We have also demonstrated that ASP act as antioxidants to scavenge superoxide, peroxynitrite, and hypochlorite and also to induce quinine reductase and stabilize LDL conformation during oxidation in vitro [25,26]. However, to date, there is no clinical evidence on the antioxidant action of ASP. Thus, the objective of this study was to examine the effect of ASP on plasma oxidative stress and redox status in a randomized crossover trial with seven healthy older adults consuming ASP in skim milk. Furthermore, the bioavailability of flavonoids in ASP was examined. 

## 2. Materials and Methods

### 2.1. Chemicals

The following reagents were obtained from Sigma Co. (St. Louis, MO, USA): catechin, kaempferol, isorhamnetin, naringenin, quercetin, butylated hydroxytoluene (BHT), Folin–Ciocalteu’s phenol reagent, copper sulfate, ethylenediaminetetraacetic acid (EDTA), β-glucuronidase type H-2 (containing sulfatase from *Helix pomatia*), glutathione reductase (type III), nicotinamide adenine dinucleotide phosphate (NADP), sodium 1-octanesulfonate monohydrate, reduced glutathione, oxidized glutathione, sodium azide, sodium chloride, sodium phosphate monobasic, sodium phosphate dibasic, 1,1,3,3-tetraethoxypropane, 2’,3’,4’-trihydroxyacetophenone, Tris buffer, magnesium chloride, triphenylphosine, and α-tocopherol. Diisopropylethylamine (DIPEA), O-bis (trimethylsilyl) trifluoroacetamide (BSTFA), and pentafluorobenzyl bromide (PFBBr) were purchased from Thermo (Boston, MA, USA). Deuterated prostaglandin (PG) F_2α_ (internal standard), PGF_2β_, and 8-iso-PGF_2α_ were purchased from Cayman (Ann Arbor, MI, USA). All organic solvents, glacial acetic acid, and potassium bromide were purchased from Fisher Co. (Fair Lawn, NJ, USA). 

### 2.2. Preparation of ASP

Almond skin powder was generously provided by the Almond Board of California. Polyphenols in 150 and 300 g almond skin powder were extracted sequentially twice using a ratio of 0.5:10 (w/v) with acidified ethanol solution (white vinegar/H_2_O/200 proof ethanol at 1/19/80) over 16 h at 4 °C. The extraction solution was selected with consideration of the extract for human use. The resulting solution was centrifuged at 1000× *g* for 15 min at 4 °C, followed by ethanol removal using a Buchi R215 Rotary Evaporator equipped with a Vacuum Controller V-850 (Flawil, Switzerland). The total phenol content in the resulting aqueous syrup was determined by Folin–Ciocalteu’s assay [27] and expressed as gallic acid equivalents (GAE). The GAE content produced from 150 and 300 g almond skin powder was 225 and 450 mg, respectively. ASP were stored overnight at 4 °C before administration to the volunteers the following morning. 

### 2.3. Subjects

Seven generally healthy older subjects (3 male, 4 female) aged 63.3 ± 9.1 years (mean ± SEM) were recruited from Boston metropolitan area by the Metabolic Research Unit (MRU) of the Jean Mayer USDA Human Nutrition Center on Aging (HNRCA) at Tufts University. All study participants completed the trial. The mean baseline lipid profile values of the seven subjects were as follows: total cholesterol (TC), 206 ± 18; triglycerides (TG), 94 ± 13; low-density lipoprotein-cholesterol (LDL-C]), 135 ± 14; high-density lipoprotein-cholesterol (HDL-C) 52 ± 7 mg/dL. Mean plasma vitamin A, C, and E concentrations were 57 ± 14 µg/dL, 0.9 ± 0.1 mg/dL, and 978 ± 123 µg/dL, respectively. Mean body weight of participants was 71 ± 6 kg; body mass index (BMI) was 25.4 ± 1.3 kg/m^2^; and systolic and diastolic blood pressure values were 128 ± 8 and 74 ± 6 mmHg, respectively. All participants were in good health and had no evidence of chronic disease on the basis of a medical history questionnaire, physical examination, electrocardiogram test, and results within normal limits of standard clinical laboratory tests, as well as fulfilling the following eligibility criteria: (1) no history of CVD, hepatic, gastrointestinal, or renal disease; (2) no alcoholism; (3) no use of antibiotics; (4) no use of supplemental multivitamins or minerals for ≥4 weeks (3 months for 60 mg vitamin C, 30 IU vitamin E, and/or 70 µg selenium) before the start of the study; and (5) no recent history of smoking. The study was approved by and performed under the guidelines of the Institutional Review Board at Tufts University Health Sciences Campus (project code: #5822). Informed consent was obtained from each subject before any study procedures were performed. Subjects were instructed not to consume alcohol or take other medications for 1 week prior to all study visits. They were also asked to consume a low-flavonoid diet for 1 week before study visits, according to the low-flavonoid diet guideline designed by the study dietitian, in which all berries, apples, pears, citrus fruits, fruit juices, onions, chocolate, wine, coffee, any kind of tea, beans, nuts, soy products, and most spices were excluded from their daily diets.

### 2.4. Study Design

The study design was a placebo-controlled, three-way crossover trial with a 1-week washout period between study visits. Subjects were randomly assigned to consume 360 mL skim milk (C), or 225 mg (L) or 450 mg (H) GAE ASP in skim milk. Although ASP were not completely soluble in skim milk, residual ASP in the glass were rinsed with water and then completely consumed. Each subject was admitted to the MRU in the morning after a 12-h overnight fast. Following a check of vital signs, an intravenous catheter was inserted into an antecubital vein of one forearm and a baseline blood sample was obtained. After subjects drank the test beverage in ~5 min, blood samples were collected at 15, 30, and 45 min and 1, 2, 3, 5, and 10 h. Lunch and dinner meals designed to contain low flavonoids and meet the recommended dietary allowances for protein and energy [28] were prepared under the supervision of the study dietitian. The same meals were served during each visit. These meals were provided at 4 and 9 h after the ASP consumption. The consumption of water, salt, sugar, and ginger ale was not limited but food and other beverages were not allowed during the intervention.

### 2.5. Sample Collection and Storage

Blood was collected into EDTA vacutainers and processed within 10 min. Whole blood was centrifuged at 1000× *g* at 4 °C for 15 min using a Sorvall RT6000 (Du Pont Co. Newtown, CT, USA). Plasma aliquots of 1.5 mL were flushed with N_2_, stored at 4 °C, and used for the Cu^2+^-induced LDL oxidation assay within 3 days. An aliquot of plasma was treated with an equivalent volume of 10% trichloroacetic acid, vortexed, and then centrifuged at 14,000× *g* for 10 min at 4 °C; then the resulting supernatant was snap-frozen for determination of reduced glutathione (GSH) and oxidized GSH (GSSG). Aliquots of plasma were snap frozen and stored at −80 °C until analysis of flavonoids, malondialdehyde (MDA), glutathione peroxidase (GPx), and F_2α_-isoprostanes. 

### 2.6. Analysis of Plasma Flavonoids

Flavonoids in plasma were determined using the HPLC method of Chen et al. [24]. Briefly, plasma was first mixed with vitamin C-EDTA solution, internal standard (2’,3’,4’-trihydroxyacetophenone), and β-glucuronidase/sulfatase. After incubation at 37 °C for 45 min, flavonoids were extracted with acetonitrile. After centrifugation, supernatant was transferred, dried under purified N_2_, and reconstituted in aqueous HPLC mobile phase for HPLC analysis. Flavonoids were determined by an HPLC system equipped with a Zorbax ODS C18 column (4.6 × 150 mm, 3.5 μm) and the Coularray 5600 A detector (ESA, Inc. Chelmsford, MA, USA). The quantification of plasma catechin, quercetin, naringenin, kaempferol, and isorhamnetin was calculated according to calibration curves constructed with authentic standards, with linear relationships of *R*^2^ > 0.999. The limit of detection on column for flavonoids was 0.5 pmol. The coefficient of variation (CV) values of intra- and inter-day assays were 3.0% and 9.0%, respectively. The recovery rate for the internal standard was 97.0 ± 0.1%.

### 2.7. Biomarkers of Antioxidant Capacity and Lipid Peroxidation

Blood for plasma glutathione analysis was collected using a drip approach from the catheter to avoid potential hemolysis which increases plasma GSH through contamination from red blood cell GSH. Blood from the catheter collected into an EDTA vacutainer. GSH and GSSG in the supernatant collected from acidified plasma were determined using the high-performance liquid chromatography with electrochemical detection (HPLC-ECD) method of Chen et al. [28]. The concentrations of plasma GSH and GSSG were calculated based on calibration curves of authentic GSH and GSSG. The intra- and inter-day assay CV values for GSH were 2.7% and 2.9% and for GSSG the values were was 6.7% and 8.1 %, respectively.

Oxygen radical absorbance capacity (ORAC) in heparinized plasma-treated 0.5 M perchloric acid (PCA) (1:1 v/v) was determined according to the method of Ou et al. [29]. The assay provides an integrated and quantitative determination of “total antioxidant capacity” by employing the area under the curve (AUC) of the magnitude and time to the oxidation of fluorescein due to peroxyl radicals generated by the addition of 2,2’-azobis (2-amidinopropane) dihydrochloride (AAPH). ORAC values were calculated according to the method described by Cao et al. [30] and are expressed as µmol/L Trolox equivalents (TE).

Plasma GPx activity was determined using the spectrophotometric method of Pleban et al. [31] with a Cobas Fara II centrifugal analyzer (Roche Diagnostics, Nutley, NJ, USA). This assay measures GPx activity on the basis of the oxidation of GSH to GSSG and the reduction of H_2_O_2_ to H_2_O, which is coupled to the oxidation of NADPH by glutathione reductase. The intra- and inter-assay CV values were 3.4% and 3.2%, respectively. 

The LDL resistance against Cu^2+^-induced oxidation was determined according to the method of Chen et al. [25]. Briefly, LDL particles were collected using an ultracentrifugation protocol. LDL (182 nmol/L) was oxidized by 10 µmol/L CuSO_4_ with (or without) in vitro addition of a final concentration of 6 µmol/L α-tocopherol in a total volume of 1.0 mL phosphate buffer. The addition of α-tocopherol was intended to amplify any potential antioxidant effect of absorbed ASP on LDL resistance against oxidation [24]. Formation of conjugated dienes was monitored by absorbance at 234 nm at 37 °C using a UV1601 spectrophotometer (Shimadzu Corp, Kyoto, Japan). The results of the LDL oxidation are expressed as lag time (defined as the intercept at the abscissa in the diene-time plot). The intra- and inter-assay CV was 1.8% and 7.5%, respectively. 

Plasma MDA was determined by the HPLC method of Volpi and Tarugi [32], in which a thiobarbituric acid-MDA conjugate product was separated by a C18 column and fluorometrically quantified at an excitation of 515 nm and emission of 553 nm. Plasma MDA concentration was calculated based on calibration curves of authentic standard 1,1,3,3-tetraethoxypropane, with a linear relationship of *R*^2^ > 0.995. The intra- and inter-assay CV values were 3.9% and 12.3%, respectively.

Plasma F_2α_-isoprostanes were measured by gas chromatograph-mass spectrometry (GC-MS) with negative chemical ionization, as described by Walter et al. [33]. Briefly, plasma lipids were isolated with Folch extraction, followed by gentle alkaline saponification to release isoprostanes. Isoprostanes were then converted to pentafluorobenzyl esters using PFBBr and DIPEA. The resulting PFB esters of F_2α_-isoprostanes were isolated using an HPLC system equipped with an amino column, dried under N_2_, and silylated with BSTFA and DIPEA. The silylated product was dried, resuspended in undecane, and analyzed by GC-MS. The final data are expressed as ng/mL. 

### 2.8. Statistical Analysis

All results are reported as mean ± SEM. Percent change from the respective baseline (0 h) value of each visit was calculated to construct the area under the curve (AUC) using the linear trapezoidal integration [34]. All percent change and AUC data were normalized by a log_10_ conversion before statistical analysis. The mixed model procedure (PROC GLM) was used to test the effects of ASP dose, time point, and their interaction on study outcomes, followed by the Tukey–Kramer honestly significant difference (Tukey’s HSD) test. Either paired *t*-test or one-way ANOVA followed by Tukey’s HSD test was employed to test the difference between the high ASP dose and control in the AUC data of MDA, F_2α_-isoprostanes, GSH, GSSG, GSH/GSSG ratio, lag time of LDL oxidation, GPx activity, and ORAC_pca_. Differences with *p* ≤ 0.05 were considered significant. The SAS 9.2 statistical software package (SAS Institute Inc. Cary, NC, USA) was used to perform all statistical analyses. 

## 3. Results

### 3.1. Bioavailability of Flavonoids

**Figure 1:**
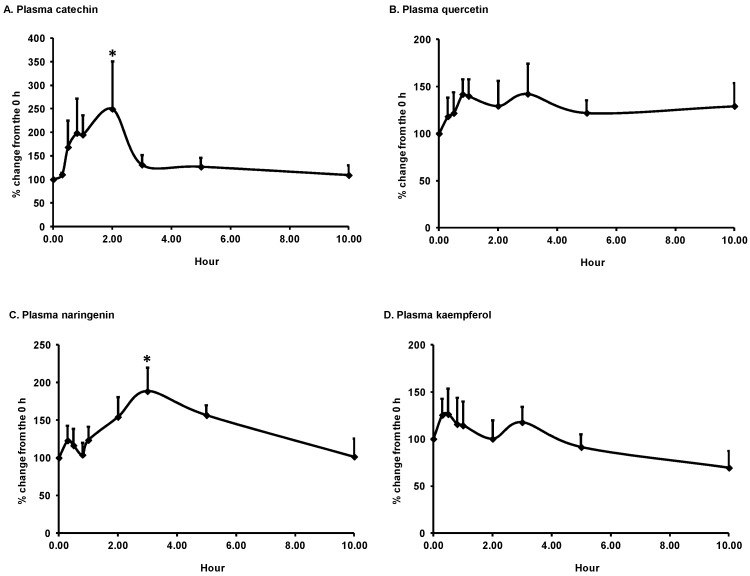

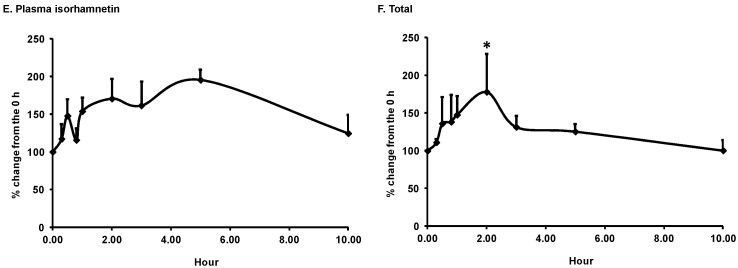
Time course of plasma flavonoids, catechin (**A**), quercetin (**B**), naringenin (**C**), kaempferol (**D**), isorhamnetin (**E**), and total (**F**) in older adults after acute intake of skim milk vehicle (C) and 450 (H) mg GAE ASP. The data are presented as the percent change from the respective baseline (0 h). The baseline concentrations of catechin, quercetin, naringenin, kaempferol, isorhamnetin, and total were 17.0 ± 4.0, 7.3 ± 1.2, 11.5 ± 5.7, 10.1 ± 3.2, 2.3 ± 0.4, and 48.1 ± 9.3 ng/mL, respectively. Values are expressed as mean ± SEM, *n* = 7. Means with a mark are significantly different from that of the baseline, *p* ≤ 0.05, tested using PROC GLM, followed by Tukey’s honestly significant difference (HSD) multi-comparison test. ASP: almond skin polyphenols; GAE: gallic acid equivalents.

Five flavonoids were quantified in the plasma of the subjects consuming H-ASP (450 mg GAE) or milk vehicle only. The baseline plasma concentration of catechin, quercetin, naringenin, kaempferol, isorhamnetin, and total flavonoid were 17.0 ± 4.0, 7.3 ± 1.2, 11.5 ± 5.7, 10.1 ± 3.2, 2.3 ± 0.4, and 48.1 ± 9.3 ng/mL, respectively. Milk alone did not significantly affect their values. The H dose led to significant increases in plasma catechin, naringenin, and sum of five flavonoids, as compared to the corresponding baseline value (*p* ≤ 0.05). Their maximum concentrations (C_max_) were 44.3 ± 15.6, 19.3 ± 8.2, and 82.3 ± 17.6 ng/mL and times to reach C_max_ (T_max_) were 1.4 ± 0.2, 3.3 ± 0.5, and 1.7 ± 0.3 h, respectively (Figure 1). At 10 h, flavonoid concentrations returned to their respective baseline values. The other three measured flavonoids, quercetin, kaempferol, and isorhamnetin, were not significantly increased by the H-ASP. 

### 3.2. Changes in Plasma Biomarkers of Antioxidant Defense

**Figure 2:**
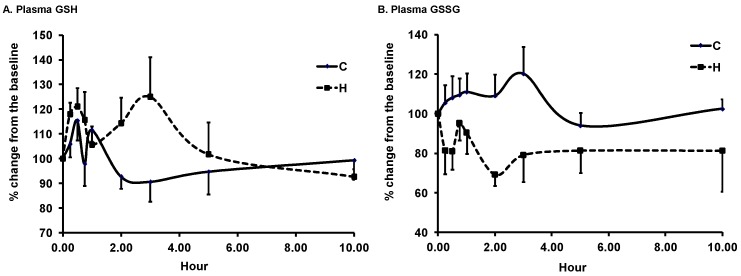

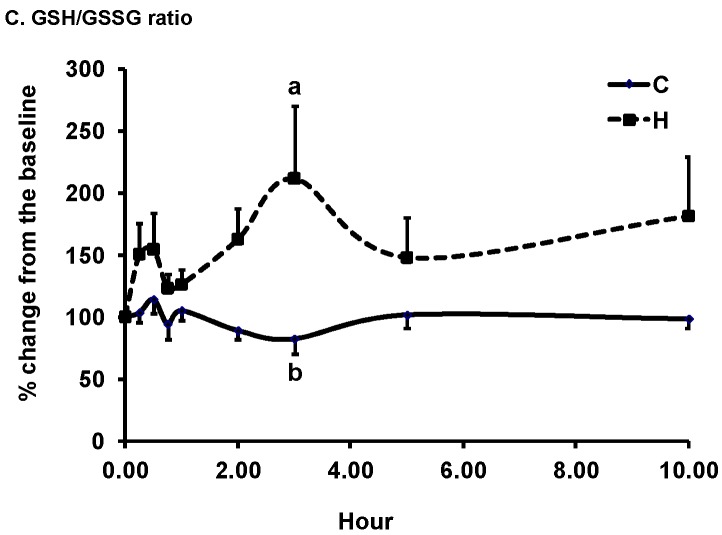
Time course of plasma glutathione (GSH) (**A**) and oxidized GSH (GSSG) (**B**) and their ratio (**C**) in older adults after acute intake of skim milk vehicle (C) and 450 (H) mg GAE ASP. The data are presented as the percent change from the respective baseline (0 h). Baseline values of GSH, GSSH, and their ratio were 1.16 ± 0.14, 0.11 ± 0.01 µmol/L, and 12.1 ± 2.4, respectively. Values are expressed as mean ± SEM, *n* = 7. Means not sharing the same letter at the same time point are significantly different, *p* ≤ 0.05, tested using PROC GLM, followed by Tukey’s HSD multi-comparison test. *p*-values of the dose effect for GSH, GSSG, and their ratio were 0.029, 0.013, and 0.014, respectively.

Mean baseline plasma values of GSH, GSSG, and their ratio were 1.16 ± 0.14, 0.11 ± 0.01 µmol/L, and 12.1 ± 2.4, respectively. A marked inter-individual variation in GSH, GSSG, and GSH/GSSG was noted. After consumption of skim milk, GSH values tended to decrease, GSSG values tended to increase, and the ratio remained unaltered (Figure 2). H-ASP tended to increase GSH by 25% at 3 h as compared to the respective baseline value and to decrease GSSG by 31% at 2 h. A favorable effect of H-ASP on GSH status was noticeable at 15 min post-consumption but retreated at 45 min and 1 h before the next favorable changes occurred at 2 and 3 h. At 3 h, the GSH/GSSG ratio of H-ASP was 212% of the baseline, which was significantly different from 82% of C at the same time point (*p* = 0.0033). The increased ratio at 3 h after H-ASP consumption was driven by both the increased GSH and the decreased GSSG. The AUC of GSH and GSSG did not differ between C and H-ASPs; the AUC of the GSG/GSSG ratio was significantly increased by 70% by H-ASP as compared to C (Table 1).

**Figure 3:**
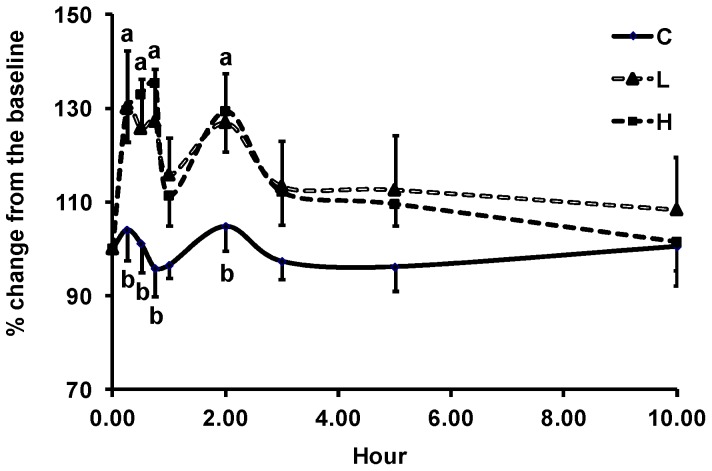
Time course of the percent change of plasma GPx activity in older adults after acute intake of skim milk vehicle (C), 225 mg (L), or 450 mg (H) GAE ASP. The data are presented as the percent change from the respective baseline (0 h). The mean baseline activity was 197.0 ± 10.6 U/L. Values are expressed as mean ± SEM, *n* = 7. Means not sharing the same letter at the same time point are significantly different, *p* ≤ 0.05, using PROC GLM followed by Tukey’s HSD multi-comparison test. *p*-Values for dose and time effect and their interaction were 0.039, ≤0.001, and 0.001, respectively.

Mean baseline plasma GPx activity was 197 ± 11 U/L. Skim milk did not affect GPx activity. L- and H-ASP up-regulated GPx activity in a two-phase mode with an initial increase occurring between 15 and 45 min, followed by the second one at 2 h (Figure 3). The effect of ASP on GPx activity was independent of the dose. The magnitude of the ASP-induced increase in GPx activity ranged between 26% and 35% from the baseline at 15, 30, and 45 min and 2 h, while skim milk slightly increased the activity during the same period. The AUC of GPx activity did not differ among three treatments (Table 1). Plasma ORAC value was not affected by ASP and milk up to 10 h. Mean baseline ORAC_pca_ value was 896 ± 28 µmol/L TE (Table 1).

### 3.3. Changes in LDL Resistance to Oxidation

**Figure 4:**
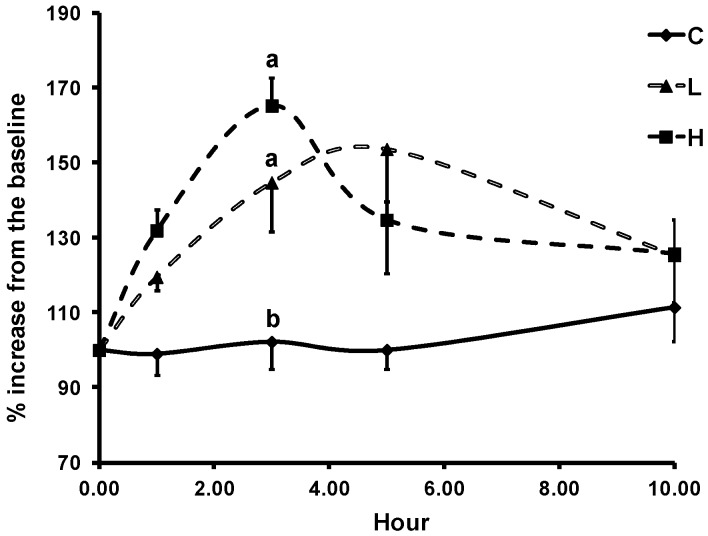
Time course of lag time of LDL oxidation with in vitro addition of 6 µmol/L α-tocopherol in older adults after acute intake of skim milk vehicle (C), 225 mg (L), or 450 mg (H) GAE ASP. The data are presented as the percent change from the respective baseline (0 h). The mean baseline lag time was 95.7 ± 2.6 min. Values are expressed as mean ± SEM, *n* = 7. Means not sharing the same letter at the same time point are significantly different, *p* ≤ 0.05, tested using PROC GLM followed by Tukey’s HSD multi-comparison test. *p*-Values of the dose and time effect and their interaction were 0.001, 0.014, and 0.049, respectively.

The mean baseline lag time of LDL oxidation was 45.1 ± 1.7 min, and its value was not extended by either ASP dose. The AUC of lag time was comparable between treatments. The addition of 6 µmol/L α-tocopherol prior to the initiation of Cu^2+^-induced LDL oxidation increased lag time to 95.7 ± 2.6 min at baseline (Figure 4). At 3 h, the lag time with added α-tocopherol after intake of L- and H-ASP was 144.7 ± 13.1 and 165.2 ± 25.0% of that at baseline, respectively, as compared to the 102.2 ± 2.4% observed after the skim milk (*p* ≤ 0.05). There was no difference in the lag time between the two ASP doses. To reduce the influence of the variation of the baseline values, the percentage change from the baseline was calculated to assess the change obtained with in vitro addition of α-tocopherol.

### 3.4. Changes in Plasma Biomarkers of Oxidative Stress

The mean baseline plasma MDA value was 2.4 ± 0.3 µmol/L. Skim milk and H-ASP did not affect MDA up to 10 h (data not shown) or its AUC (Table 2). Mean baseline plasma F_2α_-isoprostanes were 5.0 ± 0.2 ng/mL. Given that there were marked inter-individual variations, F_2α_-isoprostanes in plasma (data not shown) and their AUC (Table 2) were not affected by skim milk and H-ASP. 

## 4. Discussion

From production and processing to storage, retailing, and consumption, waste is produced in all the phases of food life cycle [10]. According to the Food and Agriculture Organization of the United Nations [35], approximately one-third of the food produced in the world for human consumption is lost or wasted annually. For example, ~30% of nonedible products of vegetables and some fruits, mainly skins and seeds, are commonly wasted and discarded [9]. Similarly, almonds, the most commonly consumed tree nut, can generate ≥4% waste as skin during the production of blanched almonds. This type of food waste has historically been used as low-value livestock feed or compost. More recently, the agri-food industry has made substantial progress in utilizing waste by-products for development of novel ingredients or products [36,37]. Successful examples include the recovery of oil from olive kernel, the production of essential oils, flavonoids, and pectin from citrus peel, and the recapture of protein concentrates from cheese whey [12]. Some of these plant-based wastes contain a variety of phytochemicals, particularly polyphenols, which are often abundant in skins and seeds [9]. For example, almond skins contain an array of flavonoids and phenolic acids, with isorhamentin-3-rutinoside being the main polyphenol [13,17]. The result of several experimental studies suggests the potential for utilizing this by-product of almond processing as a value-added ingredient useful in the development of functional foods or nutraceuticals because of its antioxidant, anti-microbial, anti-viral, neuroprotective, photoprotective, and/or prebiotic activities [13,14,15,23,24,38,39,40,41,42]. Within the last decade and with availability of new equipment, the almond blanching process has evolved to use substantially less water and more steam, reducing the loss of polyphenols into the blanch water and allowing a higher polyphenol content of the almond skin after processing. We demonstrate here the acute bioavailability and antioxidant actions of polyphenols derived from almond skins in humans without apparent untoward side effects. 

Flavonoid bioavailability is dependent on a wide array of factors, e.g., type of flavonoid, food matrix, co-consumed food components, polymorphism of detoxification mechanisms, and aging. Using a simulated human digestion system, Mandalari et al. [43] demonstrated that ASP were bioaccessible for the absorption in the upper gastrointestinal tract. In this study, the subjects consumed ASP at the dose of either 225 mg or 450 mg GAE, and plasma flavonoids were monitored over 10 h. As previously characterized in almonds, isorhamnetin is one of its principal flavonoids [13,17]; however, we found only a modest trend toward an increase in its plasma concentration. This result is in contrast with the marked increase we found in our hamster study [24]. Nonetheless, our inability to detect isorhamnetin in plasma is consistent with the report by Bartolomé et al. [44] who found it undetectable at 2.5 h post consumption of 884 mg GAE total phenols of almond skin extract. The dose employed in that study was about twice our highest dose. Bartolomé et al. study [44] estimated this dose is ~8-times higher than the dietary intake (102–121 mg/person/d) of nut polyphenols in the Spanish diet. While reports on the clinical pharmacokinetics of isorhamnetin following the consumption of isorhamnetin-rich foods or supplements are limited, Schulz et al. [45] observed a significant increase in plasma isorhamnetin with C_max_ in the 10-ng/mL range in 18 healthy men consuming one dose of 900 mg dry extract of St. John’s wort. Besides isorhamnetin, we monitored catechin, naringenin, kaempferol, and quercetin values and found significant increases in plasma catechin and naringenin with the T_max_ at 2 and 3 h, respectively. Similarly, Garrido et al. [46] reported that consumption of almond skin extract containing 884 mg GAE total phenols increased urinary excretion of epicatechin and naringenin conjugates derived from phase II metabolism. We also noted a marked inter-individual variation in the concentration of these flavonoids, which is consistent with a range of other reports that ascribe this phenomenon to the wide range of endogenous and exogenous factors mentioned above [47,48]. We did not examine urinary flavonoids or phenolic acids derived from bacteria-catalyzed flavonoids in this study. However, Llorach et al. [49] noted a plethora of phenolic acids in urine of 24 people consuming an ASP extract. This study underscores the significant role of gut microbiota on the catabolism of flavonoids in the formation of phenolic acids from flavonols, e.g., hydroxyphenylvaleric, hydroxyphenylpropionic, and hydroxyphenylacetic acids. Together with low concentrations of detected flavonoids in plasma, future research is warranted to examine the effect of phenolic acids derived from polyphenols via catabolism of colonic microflora on human health. 

Flavonoids are regarded as strong antioxidants, acting via scavenging (reducing) reactive oxidants, chelating transient metals, and/or modulating endogenous antioxidant defense mechanisms. However, the efficacy of such mechanisms of action post absorption has been questioned because of the low concentrations of flavonoids in blood and tissues as compared to other abundant endogenous antioxidants [50]. Previously, we demonstrated in hamsters that absorbed ASP enhance LDL resistance against ex vivo Cu^2+^-induced oxidation [24] and worked with the in vitro addition of vitamin E to further bolster the LDL resistance. Instead, we found the effect of the absorbed ASP on the protection of LDL against oxidation was only unmasked when α-tocopherol (in a dose-dependent manner) was added in a physiologically relevant concentration. Thus, absorbed ASP may incorporate into LDL particles and then exert antioxidative actions or/and stabilize LDL structure to enhance LDL resistance. This speculation is also based on our in vitro study showing sand α-tocopherol work in a synergistic manner to stabilize LDL conformation during oxidation [25]. While the magnitude of polyphenol bioavailability and circulating flavonoid metabolites vary between species, this study extends the putative benefit of ASP on LDL resistance to oxidation from hamsters to humans.

Flavonoids are regarded as a class of beneficial phytonutrients. However, they also display characteristics as xenobiotics and are subject to phase I, II, and III detoxification metabolism known to be involved in drug clearance from the body. In addition, they may modulate the expression of cytochrome P450 monooxygenases, phase II conjugation enzymes, and/or on membrane transporters [51]. These actions are likely attributed to the effect of flavonoids on activating xenobiotic response elements and/or antioxidant/electrophil response elements (AREs/EpREs) [51,52]. For example, via these signal transduction pathways, quercetin enhanced the expression and activities of GSH reductase, GPx, and catalase [53,54]. We determined plasma GSH and GPx activity in support of the effect of ASP on endogenous antioxidant defense systems, a result that could implicate the activation of ARE by the absorbed ASP. We found that the high-dose ASP tended to increase plasma GSH between 1 and 5 h after consumption as compared to the skim milk vehicle, suggesting the absorbed ASP either increased its production in the liver via up-regulating γ-glutamylcysteine synthetase [51] or decreased its utilization or excursion. Further, the GSH/GSSG ratio, regarded as an index of redox status, was elevated at 3 h post-consumption of the high-dose ASP as compared to the skim milk vehicle, as well as the significantly larger AUC of the ratio, substantiating the absorbed ASP could boost antioxidant defense capacity. Similarly, there was an early, transient effect of the absorbed ASP on plasma GPx activity. Although GSH is the substrate for the GPx reaction in the reduction of H_2_O_2_ and lipid peroxides to water and alcohols, we did not find a correlation between plasma GPx activity and GSH, GSSG, or their ratio (data not shown). In a previous human study, we also found that polyphenol avenanthramides from oats enabled a similar effect on GSH and GPx in humans [28]. However, neither blackberry nor cranberry juice, both rich in anthocyanins, affected erythrocyte GPx activity [55,56]. Thus, the effect of polyphenols on GPx activity and GSH status appears to depend on sample type (plasma vs. erythrocytes), flavonoid class (anthocyanins vs. flavonols), and study design (chronic vs. acute). Establishing an efficient extraction protocol to produce a commercial, high-quality polyphenol-rich product from almond skins for human use remains a challenge, though use of almond skin powder can already be found in the marketplace [57].

There are some limitations to this study, including its small sample size and absence measures of the oxidative modification of DNA and proteins. The inclusion of only healthy older adults also limits the generalizability of the results. There were significant inter-individual temporal differences in the pattern changes of plasma flavonoids and biomarkers of oxidative stress. This common phenomenon suggests marked individual genetic variation in enzymes that influence polyphenol absorption, metabolism, disposition, and excretion. Future studies are warranted to better understand interactions between genomics and flavonoid metabolism and utilization and their impact on biomarkers of oxidative stress and health outcomes.

## 5. Conclusions

During the production of blanched almonds, almond skins are generated as a waste by-product and commonly utilized as livestock feed or fertilizer. Like other waste produced during food processing, almonds skins (as well as skins from peanuts, grapes, and oranges) contain a variety of phytochemicals with a potential for development of nutraceuticals and functional foods [9]. A growing body of the literature has revealed that phytochemicals, including polyphenols and fiber in almond skins, display properties associated with health benefits, including antioxidant, anti-microbial, anti-viral, neuroprotective, photoprotective, and prebiotic activities [14,23,24,25,38,39,40,41,42]. Despite large inter-individual differences in metabolic changes, acute intake of almond skin extract increased plasma catechin, naringenin, GSH/GSSG ratio, and GPx activity at 2–3 h post-consumption. Further, the interaction of absorbed almond skin polyphenols with the in vitro addition of vitamin E unmasked the protection of the absorbed almond polyphenols on the ex vivo resistance of LDL to oxidation. In conclusion, polyphenolic constituents in blanched almond skins are capable of up-regulating antioxidant defense mechanisms and enhancing the resistance of LDL to oxidation. Thus, almond skins generated during the production of blanched almonds have the potential for use in the development of value-added ingredients and food products. 

## Figures and Tables

**Table 1:** The percent change of area under curve (AUC) of plasma glutathione, glutathione peroxidase activity, and oxygen radical absorbance capacity (ORAC_pca_) in older adults after acute intake of 250 mg (L) or 450 mg (H) almond skin polyphenols (ASP) or skim milk (C) ^1^. GPx: glutathione peroxidase.

ASP	GSH	GSSG	GSH/GSSG	GPx	ORAC_pca_^2^
	AUC (%∙h) ^3^				
C	971 ± 64	1037 ± 55	972 ± 63 ^a^	987 ± 43	1114 ± 50
L	-	-	-	1142 ± 101	1026 ± 51
H	1057 ± 67	810 ± 106	1649 ± 300 ^b^	1116 ± 47	1108 ± 33

^1^ Values are mean ± SEM, *n* = 7. ^a,b^ Means in the same column not sharing the same letter differ, *p* ≤ 0.05, using an ANOVA *t*-test, followed by Tukey’s HSD test. ^2^ Plasma was treated with perchloric acid (PCA) first before testing by ORAC assay. ^3^ Percent change from the respective baseline value (0 h) of each visit is calculated to construct the area under the curve (AUC) using the linear trapezoidal integration.

**Table 2:** The percent change of area under curve (AUC) of oxidative stress biomarkers in older adults after acute intake of 250 mg (L) or 450 mg (H) almond skin polyphenols (ASP) or skim milk (C) ^1^.

ASP	Plasma MDA	Plasma ISOPROSTANES	Lag Time of LDL Oxidation	Lag Time of LDL Oxidation with α-Tocopherol ^2^
	AUC (%∙h) ^3^			
C	954 ± 56	948 ± 125	973 ± 18	1031 ± 44 ^a^
L	-	-	1010 ± 26	1369 ± 152 ^b^
H	993 ± 70	1154 ± 198	990 ± 18	1364 ± 108 ^b^

^1^ Values are mean ± SEM, *n* = 7. ^a,b^ Means in the same column not sharing the same letter differ, *p* ≤ 0.05, using an ANOVA *t*-test, followed by Tukey’s HSD test. ^2^ α-Tocopherol (6 µmol/L) was added to LDL before the initiation of oxidation; ^3^ Percent change from the respective baseline value (0 h) of each visit is calculated to construct the area under the curve (AUC) using the linear trapezoidal integration.

## References

[1] Kamil A., Chen C.Y. (2012). Health benefits of almonds beyond cholesterol reduction. J. Agric. Food Chem..

[2] Morgillo S., Hill A.M., Coates A.M. (2019). The effects of nut consumption on vascular function. Nutrients.

[3] Neale E.P., Tapsell L.C., Guan V., Batterham M.J. (2017). The effect of nut consumption on markers of inflammation and endothelial function: A systematic review and meta-analysis of randomised controlled trials. BMJ Open.

[4] Chen G.C., Zhang R., Martinez-Gonzalez M.A., Zhang Z.L., Bonaccio M., van Dam R.M., Qin L.Q. (2017). Nut consumption in relation to all-cause and cause-specific mortality: A meta-analysis 18 prospective studies. Food Funct..

[5] Aune D., Keum N., Giovannucci E., Fadnes L.T., Boffetta P., Greenwood D.C., Tonstad S., Vatten L.J., Riboli E., Norat T. (2016). Nut consumption and risk of cardiovascular disease, total cancer, all-cause and cause-specific mortality: A systematic review and dose-response meta-analysis of prospective studies. BMC Med..

[6] International Nuts & Dried Fruits Council Nuts & Dried Fruits. https://www.nutfruit.org/consumers/news/detail/inc-2017-2018-statistical-yearbook.

[7] Grasser L.A., Fadel J.G., Garnett I., DePeters E.J. (1995). Quantity and economic importance of nine selected by-products used in California dairy rations. J. Dairy Sci..

[8] González J.F., Gañán J., Ramiro A., González-García C.M., Encinar J.M., Sabio E., Román S. (2006). Almond residues gasification plant for generation of electric power. Preliminary study. Fuel Process. Technol..

[9] Varzakas T., Zakynthinos G., Verpoort F. (2016). Plant food residues as a source of nutraceuticals and functional foods. Foods.

[10] Kumar K., Yadav A.N., Kumar V., Vyas P., Dhaliwal H.S. (2017). Food waste: A potential bioresource for extraction of nutraceuticals and bioactive compounds. Bioresour. Bioprocess..

[11] Babbar N., Oberoi H.S., Sandhu S.K. (2015). Therapeutic and nutraceutical potential of bioactive compounds extracted from fruit residues. Crit. Rev. Food Sci. Nutr..

[12] Esfahlan A.J., Jamei R., Esfahlan R.J. (2010). The importance of almond (*Prunus amygdalus* L.) and its by-products. Food Chem..

[13] Monagas M., Garrido I., Lebron-Aguilar R., Bartolome B., Gomez-Cordoves C. (2007). Almond (*Prunus dulcis* (Mill.) D.A. Webb) skins as a potential source of bioactive polyphenols. J. Agric. Food. Chem..

[14] Mandalari G., Faulks R.M., Bisignano C., Waldron K.W., Narbad A., Wickham M.S. (2010). In vitro evaluation of the prebiotic properties of almond skins (*Amygdalus communis* L.). FEMS Microbiol. Lett..

[15] Mandalari G. (2012). Potential health benefits of almond skin. J. Bioprocess. Biotech..

[16] Bolling B.W., Dolnikowski G., Blumberg J.B., Oliver Chen C.Y. (2009). Quantification of almond skin polyphenols by liquid chromatography-mass spectrometry. J. Food Sci..

[17] Milbury P.E., Chen C.Y., Dolnikowski G.G., Blumberg J.B. (2006). Determination of flavonoids and phenolics and their distribution in almonds. J. Agric. Food Chem..

[18] Ann Lila M. (2006). The nature-versus-nurture debate on bioactive phytochemicals: The genome versus terroir. J. Sci. Food Agric..

[19] Hooper L., Kroon P.A., Rimm E.B., Cohn J.S., Harvey I., Le Cornu K.A., Ryder J.J., Hall W.L., Cassidy A. (2008). Flavonoids, flavonoid-rich foods, and cardiovascular risk: A meta-analysis of randomized controlled trials. Am. J. Clin. Nutr..

[20] Kim Y., Je Y. (2017). Flavonoid intake and mortality from cardiovascular disease and all causes: A meta-analysis of prospective cohort studies. Clin. Nutr. ESPEN.

[21] Grosso G., Micek A., Godos J., Pajak A., Sciacca S., Galvano F., Giovannucci E.L. (2017). Dietary flavonoid and lignan intake and mortality in prospective cohort studies: Systematic review and dose-response meta-analysis. Am. J. Epidemiol..

[22] Bolling B.W., Chen C.Y., McKay D.L., Blumberg J.B. (2011). Tree nut phytochemicals: Composition, antioxidant capacity, bioactivity, impact factors. A systematic review of almonds, Brazils, cashews, hazelnuts, macadamias, pecans, pine nuts, pistachios and walnuts. Nutr. Res. Rev..

[23] Mandalari G., Bisignano C., D’Arrigo M., Ginestra G., Arena A., Tomaino A., Wickham M.S. (2010). Antimicrobial potential of polyphenols extracted from almond skins. Lett. Appl. Microbiol..

[24] Chen C.Y., Milbury P.E., Lapsley K., Blumberg J.B. (2005). Flavonoids from almond skins are bioavailable and act synergistically with vitamins C and E to enhance hamster and human LDL resistance to oxidation. J. Nutr..

[25] Chen C.Y., Milbury P.E., Chung S.K., Blumberg J.B. (2007). Effect of almond skin polyphenolics and quercetin on human LDL and apolipoprotein B-100 oxidation and conformation. J. Nutr. Biochem..

[26] Chen C.Y., Blumberg J.B. (2008). In vitro activity of almond skin polyphenols for scavenging free radicals and inducing quinone reductase. J. Agric. Food Chem..

[27] Singleton V.L., Orthofer R., Lamuela-Ravent R.M. (1999). Analysis of total phenols and other oxidation substrates and antioxidants by means of Folin–Ciocalteu reagent. Methods Enzymol..

[28] Chen C.Y., Milbury P.E., Collins F.W., Blumberg J.B. (2007). Avenanthramides are bioavailable and have antioxidant activity in humans after acute consumption of an enriched mixture from oats. J. Nutr..

[29] Ou B., Hampsch-Woodill M., Prior R.L. (2001). Development and validation of an improved oxygen radical absorbance capacity assay using fluorescein as the fluorescent probe. J. Agric. Food Chem..

[30] Cao G., Alessio H.M., Cutler R.G. (1993). Oxygen-radical absorbance capacity assay for antioxidants. Free Radic. Biol. Med..

[31] Pleban P.A., Munyani A., Beachum J. (1982). Determination of selenium concentration and glutathione peroxidase activity in plasma and erythrocytes. Clin. Chem..

[32] Volpi N., Tarugi P. (1998). Improvement in the high-performance liquid chromatography malondialdehyde level determination in normal human plasma. J. Chromatogr. Biomed. Sci. Appl..

[33] Walter M.F., Blumberg J.B., Dolnikowski G.G., Handelman G.J. (2000). Streamlined F2-isoprostane analysis in plasma and urine with high-performance liquid chromatography and gas chromatography/mass spectroscopy. Anal. Biochem..

[34] Nielsen I.L., Chee W.S., Poulsen L., Offord-Cavin E., Rasmussen S.E., Frederiksen H., Enslen M., Barron D., Horcajada M.N., Williamson G. (2006). Bioavailability is improved by enzymatic modification of the citrus flavonoid hesperidin in humans: A randomized, double-blind, crossover trial. J. Nutr..

[35] FAO (2011). Global Food Losses and Food Waste—Extent, Causes and Prevention.

[36] Baiano A. (2014). Recovery of biomolecules from food wastes—A review. Molecules.

[37] Rudra S.G., Nishad J., Jakhar N., Kaur C. (2015). Food industry waste: Mine of nutraceuticals. Int. J. Sci. Environ. Technol..

[38] Bisignano C., Mandalari G., Smeriglio A., Trombetta D., Pizzo M.M., Pennisi R., Sciortino M.T. (2017). Almond skin extracts abrogate HSV-1 replication by blocking virus binding to the cell. Viruses.

[39] Bisignano C., Filocamo A., La Camera E., Zummo S., Fera M.T., Mandalari G. (2013). Antibacterial activities of almond skins on cagA-positive and-negative clinical isolates of *Helicobacter pylori*. BMC Microbiol..

[40] Mandalari G., Bisignano C., Genovese T., Mazzon E., Wickham M.S., Paterniti I., Cuzzocrea S. (2011). Natural almond skin reduced oxidative stress and inflammation in an experimental model of inflammatory bowel disease. Int. J. Immunopharmacol..

[41] Mandalari G., Genovese T., Bisignano C., Mazzon E., Wickham M.S., Di Paola R., Bisignano G., Cuzzocrea S. (2011). Neuroprotective effects of almond skins in experimental spinal cord injury. Clin. Nutr..

[42] Arena A., Bisignano C., Stassi G., Mandalari G., Wickham M.S., Bisignano G. (2010). Immunomodulatory and antiviral activity of almond skins. Immunol. Lett..

[43] Mandalari G., Tomaino A., Rich G.T., Lo Curto R.B., Arcoraci T., Martorana M., Bisignano C., Saija A., Parker M.L., Waldron K. (2010). Polyphenol and nutrient release from skin of almonds during simulated human digestion. Food Chem..

[44] Bartolome B., Monagas M., Garrido I., Gomez-Cordoves C., Martin-Alvarez P.J., Lebron-Aguilar R., Urpi-Sarda M., Llorach R., Andres-Lacueva C. (2010). Almond (*Prunus dulcis* (Mill.) D.A. Webb) polyphenols: From chemical characterization to targeted analysis of phenolic metabolites in humans. Arch. Biochem. Biophys..

[45] Schulz H.U., Schurer M., Bassler D., Weiser D. (2005). Investigation of pharmacokinetic data of hypericin, pseudohypericin, hyperforin and the flavonoids quercetin and isorhamnetin revealed from single and multiple oral dose studies with a hypericum extract containing tablet in healthy male volunteers. Arzneimittelforschung.

[46] Garrido I., Urpi-Sarda M., Monagas M., Gomez-Cordoves C., Martin-Alvarez P.J., Llorach R., Bartolome B., Andres-Lacueva C. (2010). Targeted analysis of conjugated and microbial-derived phenolic metabolites in human urine after consumption of an almond skin phenolic extract. J. Nutr..

[47] Manach C., Williamson G., Morand C., Scalbert A., Remesy C. (2005). Bioavailability and bioefficacy of polyphenols in humans. I. Review of 97 bioavailability studies. Am. J. Clin. Nutr..

[48] Williamson G., Manach C. (2005). Bioavailability and bioefficacy of polyphenols in humans. II. Review of 93 intervention studies. Am. J. Clin. Nutr..

[49] Llorach R., Garrido I., Monagas M., Urpi-Sarda M., Tulipani S., Bartolome B., Andres-Lacueva C. (2010). Metabolomics study of human urinary metabolome modifications after intake of almond (*Prunus dulcis* (Mill.) D.A. Webb) skin polyphenols. J. Proteome Res..

[50] Hollman P.C., Cassidy A., Comte B., Heinonen M., Richelle M., Richling E., Serafini M., Scalbert A., Sies H., Vidry S. (2011). The biological relevance of direct antioxidant effects of polyphenols for cardiovascular health in humans is not established. J. Nutr..

[51] Moon Y.J., Wang X., Morris M.E. (2006). Dietary flavonoids: Effects on xenobiotic and carcinogen metabolism. Toxicol. In Vitro.

[52] Myhrstad M.C., Carlsen H., Nordstrom O., Blomhoff R., Moskaug J.O. (2002). Flavonoids increase the intracellular glutathione level by transactivation of the gamma-glutamylcysteine synthetase catalytical subunit promoter. Free Radic. Biol. Med..

[53] Milner J.A., McDonald S.S., Anderson D.E., Greenwald P. (2001). Molecular targets for nutrients involved with cancer prevention. Nutr. Cancer.

[54] Moskaug J.O., Carlsen H., Myhrstad M.C., Blomhoff R. (2005). Polyphenols and glutathione synthesis regulation. Am. J. Clin. Nutr..

[55] Hassimotto N.M., Pinto Mda S., Lajolo F.M. (2008). Antioxidant status in humans after consumption of blackberry (*Rubus fruticosus* L.) juices with and without defatted milk. J. Agric. Food Chem..

[56] Duthie S.J., Jenkinson A.M., Crozier A., Mullen W., Pirie L., Kyle J., Yap L.S., Christen P., Duthie G.G. (2006). The effects of cranberry juice consumption on antioxidant status and biomarkers relating to heart disease and cancer in healthy human volunteers. Eur. J. Nutr..

[57] Garrido I., Monagas M., Gomez-Cordoves C., Bartolome B. (2008). Polyphenols and antioxidant properties of almond skins: Influence of industrial processing. J. Food Sci..

